# Gal epitope expression and immunological properties in *iGb3S* deficient mice

**DOI:** 10.1038/s41598-018-33032-7

**Published:** 2018-10-18

**Authors:** Anliang Shao, Liming Xu, Xi Wu, Susu Liu, Yan Lu, Changfa Fan

**Affiliations:** 1 Institute of Medical Device Control, National Institutes for Food and Drug Control, 102629 Beijing, China; 2 Institute for Laboratory Animal Resources, National Institutes for Food and Drug Control, 102629 Beijing, China; 3 School of Medical Lab Science and Life Science, Wenzhou Medical University, 325035 Wenzhou, China; 4 Subei People’s Hospital of Jiangsu Province, 225001 Jiangsu, China

## Abstract

The Gal antigen is synthesized by glycoprotein galactosyltransferase alpha 1, 3 (*GGTA1*) or (and) isoglobotrihexosylceramide 3 synthase (*iGb3S*). However, whether *iGb3S* deletion changes Gal epitope expression and immunological properties in animals is still not clear. The objective of this study was to develop *iGb3S* deficient mice, and characterize their Gal epitope expression and Gal epitope-related immunological properties. *iGb3S* gene knockout mice were generated on the C57BL/6 background using the bacterial artificial chromosome homology region recombination technique. Gal epitope expression in the *iGb3S* deficient mice was determined by using a monoclonal anti-Gal antibody. Immunological properties were analyzed by enzyme linked immune sorbent assay. It was found that Gal epitope expression was decreased from 5.19% to 21.74% in the main organs of *iGb3S* deficient mice, compared with that of C57BL/6 wild type mice, suggesting that the *iGb3S* gene participated to Gal epitope expression. However, *iGb3S* deletion alone did not cause significant changes in the immunological properties of *iGb3S* deficient mice with or without exogenous Gal antigen (Rabbit Red Blood Cell) stimulation. The data from this study suggest that the *iGb3S* gene likely contributes to Gal epitope expression, but may have a very weak effect on immunological properties of the *iGb3S* deficient mice.

## Introduction

Many studies have shown that the major antigen in pig tissue recognized by primate antibodies is a terminal galalpha1-3gal carbohydrate structure (Gal antigen) present on glycolipids and glycoproteins^1–4^. Furthermore, anti-Gal natural antibodies are responsible for hyperacute rejection in pig-to-primate xenotransplantation^5–7^. It is known that Gal antigen is synthesized by glycoprotein galactosyltransferase alpha 1, 3 (*GGTA1*) or (and) isoglobotrihexosylceramide 3 synthase (*iGb3S*), and that *GGTA1* contributes to the glycoprotein type and *iGb3S* contributes to the glycolipidtype^8–10^. *iGb3S* mRNA was detected in mouse tissues and pig tissues^9,10^, but humans lack *iGb3S* expression except in the thymus and monocyte-derived dendritic cells^11^.

Several studies showed that the Gal epitope is expressed in *GGTA1* deficient mice (splenic fibroblasts and tissues including the pancreas, spleen, kidney and liver), and in fetal-pig homozygous *GGTA1* knockout (KO) fibroblasts when stained with anti-Gal alpha(1,3)Gal mAb or with sensitized human serum^9,12,13^. We also verified that the Gal epitope was expressed in *GGTA1* KO mice developed in our laboratory, by a standardized Gal antigen quantitative detection method using a commercial specific anti-Gal antibody (M86, mAb) [unpublished data]. Christiansen *et al*.^11^ showed that purified normal human anti-Gal immunoglobulin G can bind to iGb3 lipid to mediate complement lysis of transfected human cells expressing iGb3, suggesting that iGb3 may represent an important obstacle in xenotransplantation^11^. The results from Milland *et al*.^13^ verified that *GGTA1* KO mice have mRNA for *iGb3S* and induce an antibody response to Gal antigen synthesized by *iGb3S*^12^. Another study by Milland *et al*.^9^ showed that transfection of *iGb3S* cDNA resulted in high levels of cell surface Galalpha(1, 3)Gal synthesized via the isoglobo series pathway, thus demonstrating that mouse *iGb3S* is an additional enzyme capable of synthesizing the xenoreactive Galalpha(1, 3)Gal epitope. Anti-Gal antibody responses were induced in *GGTA1* KO mice after immunization with *GGTA1*-positive cells or *iGb3S*-positive cells, indicating *iGb3S* mediates Gal antigen mediated immunologic toxicity^9^.

However, a study by Puga Yung *et al*.^14^ demonstrated that *iGb3S* mRNA was expressed in all pig tissues tested whether derived from wild-type (WT) or *GGTA1* KO animals, but iGb3 was absent^14^. Another study showed that iGb3 or other isoglobo-series glycosphingolipids were not detected in pig organs, including the heart, liver, pancreas, and kidney, by ion-trap mass spectrometry^15^. Diswall *et al*.^16^ demonstrated that a complete lack of α-Gal glycolipid reactivity in the *GGTA1* KO pig small intestine examined with different anti-Gal reagents such as mono and polyclonal Abs and lectins^16^.

Currently, information about the relevance of *iGb3S* with Gal epitope expression is controversial, and there is a lack of data to indicate whether *iGb3S* contributes to Gal epitope expression. Some studies of *iGb3S* KO mice only focused on iNKT cell function^11,17,18^, but did not show any information about the relevance of the *iGb3S* gene with Gal epitope expression. In this study, a C57BL/6 derived embryonic stem (ES) cell line was used to establish an *iGb3S* deficient mouse. The use of the C57BL/6 background for *iGb3S* deficient model provided a pure genetic background suitable for immunological study, unlike those made with ES cell lines derived from 129/Sv mice, which need several generations of backcrossing to C57BL/6 mice to obtain a uniform genetic background. Gal epitope expression profiling in the main organs and immunological properties, including total antibody and anti-Gal antibody activity (anti-Gal IgG, IgM, and IgA) in the *iGb3S* deficient mouse were examined. The results from this study provide basic information to help understand whether the *iGb3S* gene contributes to Gal epitope expression, and the xeno-species anti-Gal antibody-mediated immune response.

## Results

### Generation of *iGb3S* KO mice

**Figure 1:**
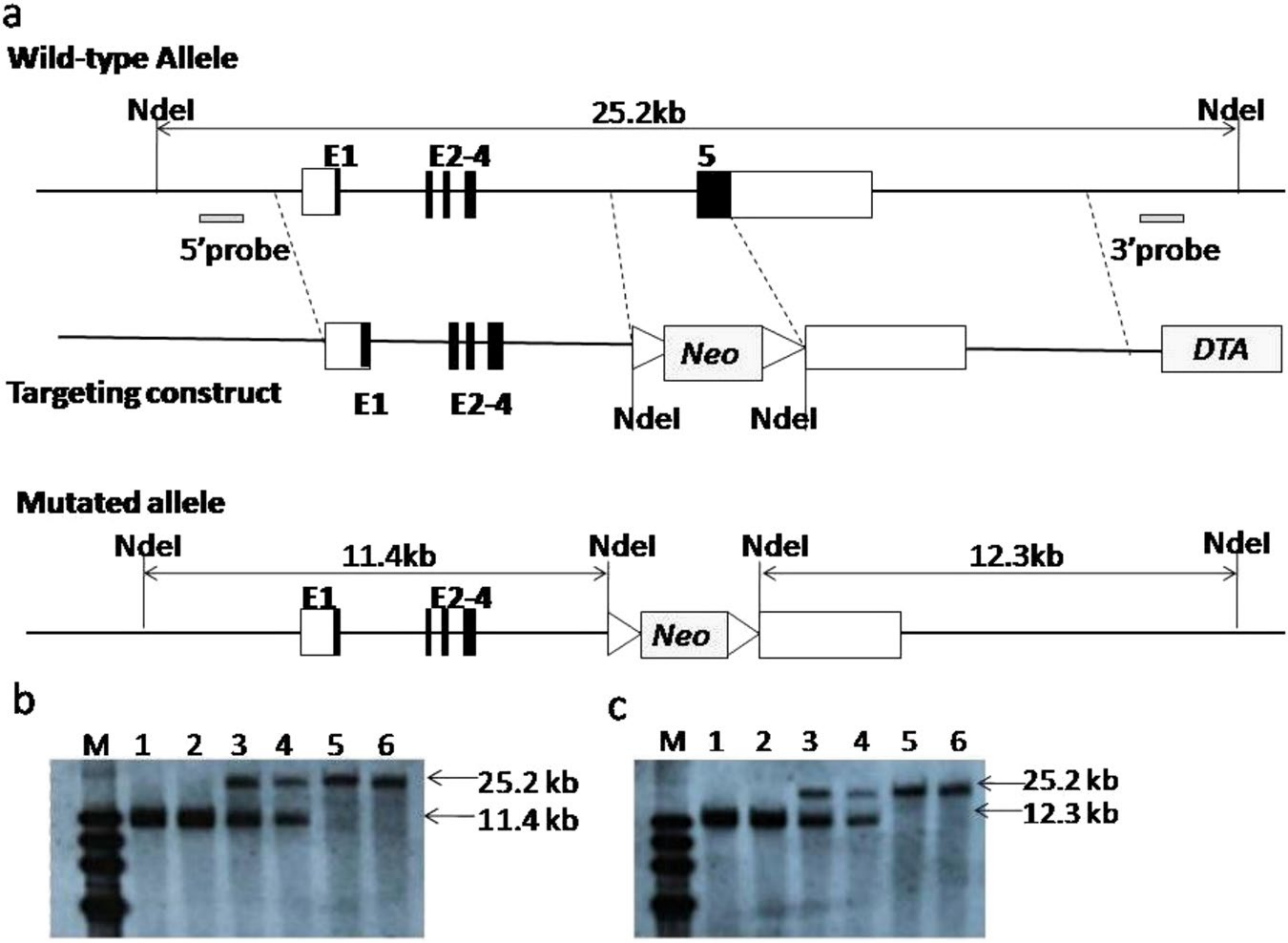
Targeting vector construction strategies. (**a**) The top graph shows the wild-type allele with exon 1 to 5 in the murine *iGb3S* WT locus, together with relevant enzyme restriction sites and probes for Southern blot analysis. The middle graph shows the targeting vector constructed by replacing a 1.5-kb fragment encoding the *iGb3S* exon 5 with a loxP-flanked neomycin-resistance gene cassette (neo), and a diphtheria toxin A (DTA). The lower graph shows the mutated allele with an11.4 kb *Nde*1-fragment at the 5′-arm immediately upstream of the neo cassette and 12.3 kb of the *Nde*1-fragment at the 3′-arm immediately downstream of the neo cassette. Coding and non-coding regions of exon 1–5 are marked by filled and non-filled boxes, respectively. Homology arms are depicted as bold lines. (**b**,**c**) show Southern blotting results. (**b**) Genomic DNA was digested with *Nde*I in the 5′-probe and resulted in 25.2 kb and 11.4 kb fragments. (**c**) Genomic DNA was digested with *Nde*I in the 3′-probe and resulted in 25.2 kb and 12.3 kb fragments. M, DNA marker; lanes 1 and 2 are mut/mut, lanes 3 and 4 are mut/wt; lanes 5 and 6 are wt/wt.

The coding sequence of the fifth exon of *iGb3S* responsible for enzymatic activity was replaced by a loxP-flanked neomycin resistance cassette (Fig. 1a). Homologous recombination at the *iGb3S* locus in C57BL/6 ES cells was confirmed by Southern blotting and 11 mutant ES cell clones were obtained (Figs S1 and S2). After microinjection of mutant ES cell clones into pseudo pregnant white mice, six chimeric *iGb3S* mice (white color with black-dot) were obtained. Heterozygous F1 progenies were obtained by breeding chimeric iGb3S mice with WT C57BL/6 mice and F1 progenies with pure black color were selected as parents for breeding and expansion. Homozygous *iGb3S* KO mice were obtained by intercrossing (Fig. S3). The homologous recombination at the *iGb3S*-locus and exon 5 deletion of *iGb3S* gene was confirmed over five generations of *iGb3S* KO mice by Southern blot analysis with 3′- and 5′-probes. As shown in Fig. 1b,c, the band of the 3′-probe 1-*Nde*I was 25.2 kb in WT mice and 11.4 kb in mutant mice, and the band of the 5′-probe 2-*Nde*I was 25.2 kb in WT mice and 12.3 kb in mutant mice. Bands 1 and 2 were from samples taken from homologous *iGb3S* KO mice. *iGb3S* KO mice reproduced normally and progeny were born at expected Mendelian ratios. They grew normally and exhibited no overt developmental or behavioral defects. Body and organ weights were not significantly different compared with WT littermates (Tables S1 and S2.). Histological examination of main organs did not reveal differences compared with WT littermates (Fig. S4). The fifth or later generations of *iGb3S* KO mice were used to detect Gal epitope expression and for the immunological properties assay.

### mRNA expression of *iGb3S* and *GGTA1* in *iGb3S* KO mice

**Figure 2:**
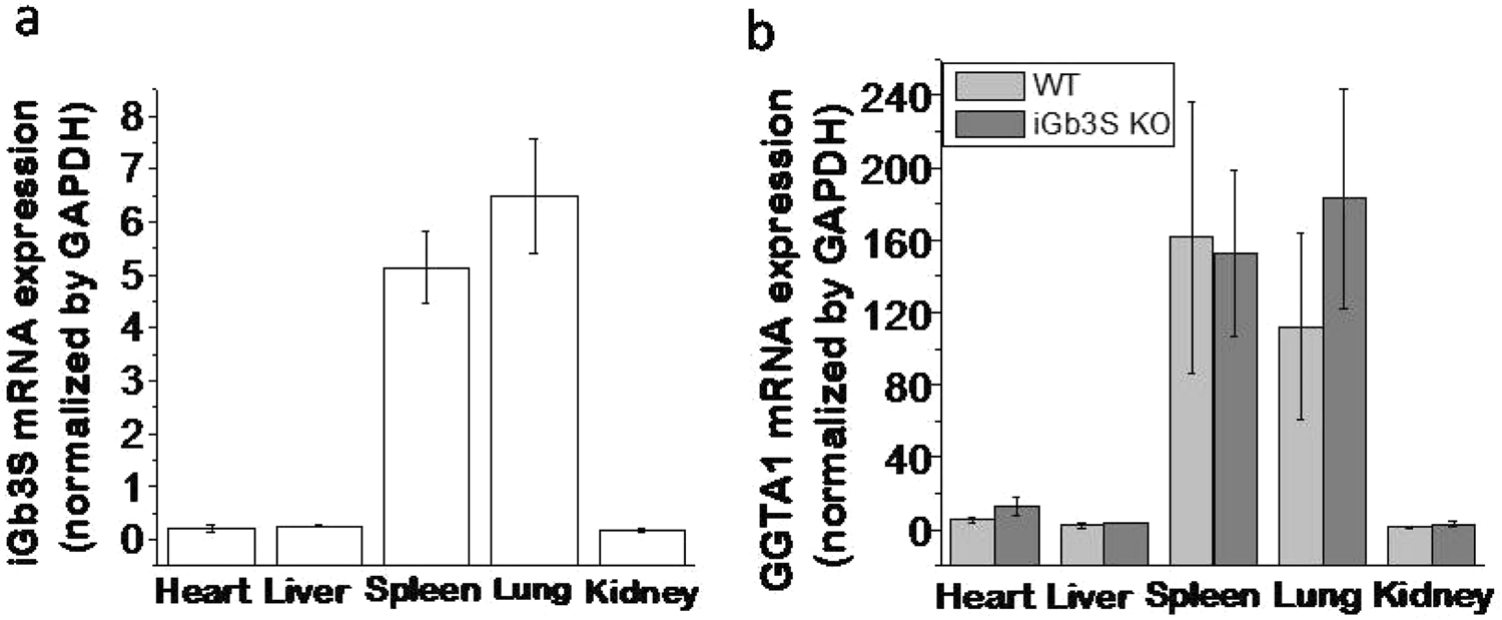
The mRNA expression level determined by RT-PCR. (**a**) *iGb3S* mRNA expression level in WT mice; (**b**) *GGTA1* mRNA expression level in WT and *iGb3S* KO mice. The relative mRNA expression level is shown as 2^−ΔCt^, and is normalized to the *GAPDH* gene. There is no significant difference at *GGTA1* mRNA expression level in *iGb3S* KO mice compared to WT mice.

*iGb3S* deficiency was confirmed at the mRNA level in selected organs of *iGb3S* KO mice by real-time polymerase chain reaction (RT-PCR). As expected, *iGb3S* mRNA expression disappeared in *iGb3S* KO mice (data not shown). To obtain more information regarding the relevance of the *GGTA1* and *iGb3S* genes in Gal antigen expression, *GGTA1* mRNA from WT (n = 5) and *iGb3S* KO mice (n = 8) was investigated. Relative *GGTA1* and *iGb3S* mRNA expressions in WT mice were strongly expressed in the spleen and lung, and weakly expressed in the heart, liver, and kidney as shown in Fig. 2a,b. No significant differences in the *GGTA1* mRNA expression in *iGb3S* KO mice were observed when compared with WT littermates (Fig. 2b).

### Gal epitope expression in *iGb3S* KO mice

Gal epitope expression in WT C57BL/6 mice was higher in the spleen and lung compared with the heart, liver, and kidney (Table 1). This distribution tendency of Gal epitope expression in different tissues was in accord with the tendency of *GGTA1* and *iGb3S* mRNA expression, implying the *GGTA1* and *iGb3S* genes correlated with Gal epitope expression. In the selected tissues of *iGb3S* KO mice (n = 8), Gal epitope expression was significantly decreased by about 16.2% in the spleen, 10.1% in the lung, and 21.74% in the liver, and was slightly decreased in the kidney (5.19%) and heart (8.31%) compared with WT C57BL/6 mice (Table 2, one-way analysis of variance, p < 0.05). These results indicated that the *iGb3S* gene participated in Gal epitope expression in mice.

**Table 1:** Gal epitope expression in the main organs of *iGb3S* KO mice and WT mice.

(n × 10^11^ Gal epitopes/mg wet tissue)					
	Spleen	Heart	Liver	Kidney	Lung
WT type mice	327.70 ± 20.74	81.70 ± 5.91	21.47 ± 3.95	48.18 ± 0.67	360.34 ± 18.37
*iGb3S* KO mice	274.61 ± 0.14^#^	74.91 ± 7.61	12.03 ± 1.38^#^	45.68 ± 0.81	323.95 ± 10.22^#^
Decrease rate (%)^*^	16.20	8.31	21.74	5.19	10.10

^*^Decreased percentage of Gal epitope expression in the main organs of *iGb3S* KO mice (n = 8) compared with WT C57BL/6 mice (n = 5); ^#^p < 0.05 compared to WT mice.

**Table 2:** Primers used in target vector construction in this study.

Primer	Sequence	Restriction Enzyme	Product size	Tm
*iGb3S*-A-F	cgatGGTACCGATATCACAGAATCTTTCTCTGTTTTC	*KpnI/EcoRV*	547 bp	53
*iGb3S*-A-R	cgatGAATTCCATATGCAGGGTTTCTCTGTGTAGCC	*EcoRI/NdeI*		56
*iGb3S*-B-F	cgatGGATCCCATATGCAGCCCTTCCCTGGCCAAGC	*BamHI/NdeI*	486 bp	64
*iGb3S*-B-R	cgatGCGGCCGCGATATCCTGGTCACAGGAATGGCTTCA	*NotI/EcoRV*		64
*iGb3S*-C-F	cgatCTCGAGGTGGATGTCTCAGTGTGCGAA	*XhoI*	586 bp	58
*iGb3S*-C-R(in)	TACCGCAGACGGTGGATATCATTGACAGTCACTGAGCAA	*EcoRV*		56
*iGb3S*-C-F(in)	TTGCTCAGTGACTGTCAATGATATCCACCGTCTGCGGTA	*EcoRV*	585 bp	56
*iGb3S*-C-R	cgatGCGGCCGCCTATCGAGTGGTTATTCTCAGGG	*NotI*		56
*iGb3S*-Atest-F	TAGAATACACACCTAATATTGATTAGCA		673 bp	54
*iGb3S*-Atest-R	TGGACGTAAACTCCTCTTCAG			55
*iGb3S*-Btest-F	Neo-F		734 bp	56
*iGb3S*-Btest-R	GGAGAGCTGAGGCTGAAGTC			58
*iGb3S*-C1test-F	M13R		730 bp	56
*iGb3S*-C1test-R	GGCTAAATGCACCTGTCATG			56
*iGb3S*-C2test-F	ACACAAGGACTTGACCATGG		667 bp	56
*iGb3S*-C2test-R	C2testR			56

### The immunological properties of *iGb3S KO* mice

**Figure 3:**
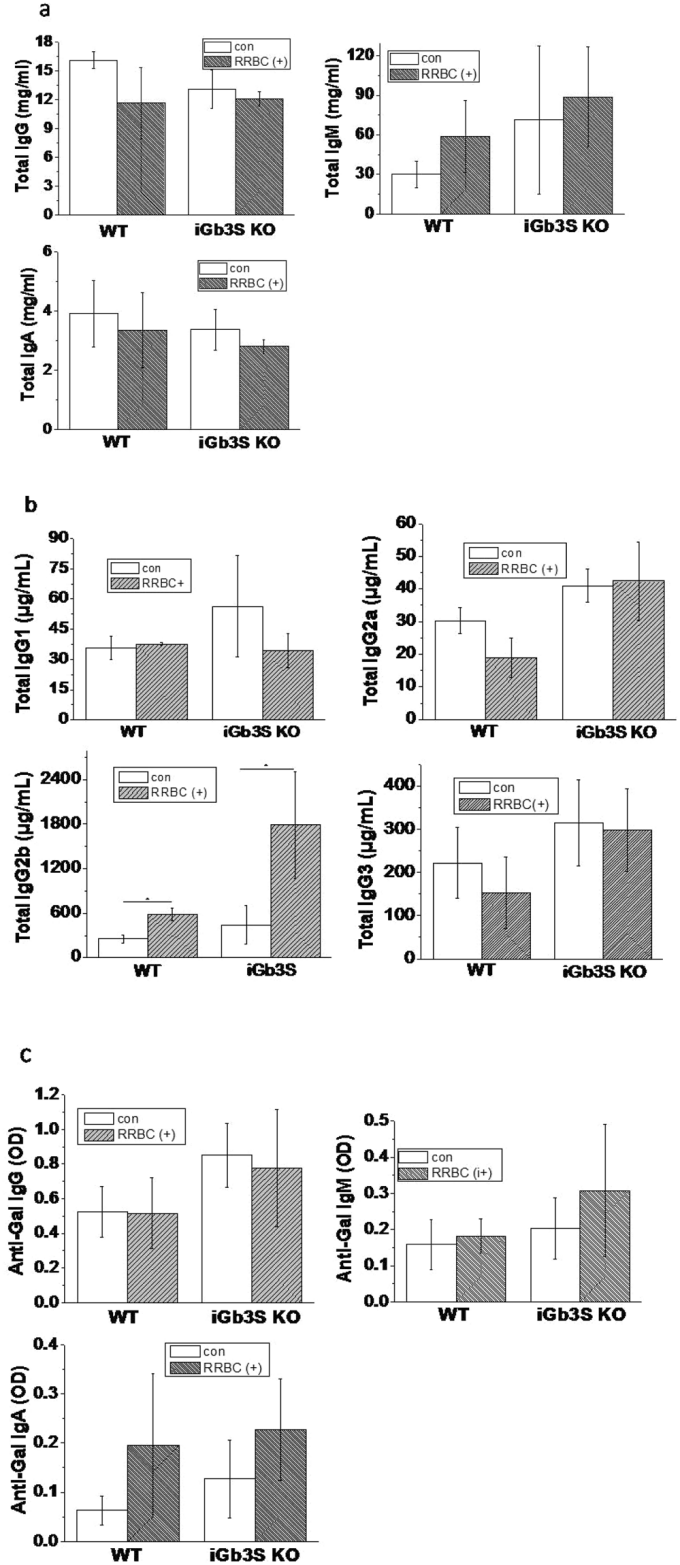
The level of immunological factors in WT and *iGb3S* KO mice with or without RRBC immunization. (**a**) Total immunoglobulin levels (IgG, IgM, and IgA); (**b**) Sub-group IgG levels; c. Anti-Gal antibody levels (anti-Gal IgG, anti-Gal IgM, and anti-Gal IgA). *p < 0.05, in mice with RRBC immunization compared to mice without RRBC immunization. Indication: Origin 8 graphics program was used to create the artwork, and save them as tiff.

To investigate the immunological properties of *iGb3S* KO mice, immunological factors were measured. Serum total IgG, IgM, and IgA expression levels are shown in Fig. 3a,b. There was no significant difference in total Ig levels between *iGb3S* KO mice with or without rabbit blood red cell (RRBC) immunization and WT mice. But, IgG2b expression was significantly increased after RRBC immunized treatment in *iGb3S* KO and WT mice (one-way analysis of variance, p < 0.05) compared with no-treatment control mice, respectively. IgG1, IgG2a, and Ig G3 expressions were not significantly different between *iGb3S* KO mice with or without RRBC immunization and WT mice.

To determine whether *iGb3S* KO mice would respond to specific Gal epitope immunization, anti-Gal IgG, anti-Gal IgM and anti-Gal IgA were assessed in *iGb3S* KO mice (Fig. 3c). Only background optical density (OD) values, similar to OD values in WT mice, were detected in *iGb3S* KO mice, and no significant differences in the OD values of anti-Gal IgG and anti-Gal IgM after RRBC immunization of *iGb3S* KO mice was observed compared with the non-treatment control group and WT mice. The OD value of IgA expression in *iGb3S* KO mice was slightly higher than that in WT mice with and without RRBC treatment, but this was not statistically significant.

## Discussion

To understand whether *iGb3S* is involved in Gal antigen expression and anti-Gal antibody responses, we generated *iGb3S* KO mice. First, it was confirmed that WT C57BL/6 mice strongly expressed *iGb3S* mRNA in the spleen and lung, and weakly in the heart, liver, and kidney (Fig. 2a). This distribution pattern of *iGb3S* mRNA in the main organs is similar to *GGTA1* mRNA expression (Fig. 2b), albeit at a lower level than *GGTA1* mRNA. *iGb3S* mRNA in *iGb3S* KO mice completely disappeared as expected, but there were no changes in *GGTA1* mRNA compared with that in WT mice (Fig. 2b). It was found that Gal epitope expression in *iGb3S* KO mice was decreased by about 5.19% to 21.74% in the main organs (Table 2), indicating that the *iGb3S* gene is likely contributes to Gal epitope expression. Previously, studies of the *iGb3S* gene have focused on the influence of the iGb3 protein on the development and function of invariant natural killer cells^11,17,18^. Here, the data showed that deletion of the *iGb3S* gene decreased Gal epitope expression, even though the data was not significance in the heart and kidney, that possibly because the Gal epitope expression was very low (near the detection limit) in these tissues, as well as large difference between individuals. Our unpublished data and the data from several previous studies demonstrated that the Gal epitope was still expressed in *GGTA1* KO mice^9,10,12,13^. Milland *et al*.^9^ showed that transfection of *iGb3S* cDNA resulted in high levels of cell surface Gal synthesized via the isoglobo series pathway, demonstrating that mouse *iGb3S* can synthesize the xenoreactive Gal epitope. Our data support that the *iGb3S* gene is another source for the synthesis of Gal antigen in mice.

A recent study demonstrated that silencing the porcine *iGb3S* gene did not affect Gal levels. However, they used IB4 staining for α-GAL/iGb3 measurements. It is known that the specificity of IB4 binding to different number of sugars in the Gal antigen is varied. A previous study showed that IB4 lectin is insufficient for the detection of relatively small numbers of Gal epitopes, because of the low binding affinity of the monomeric interaction of the lectin molecule with more than one of the four combining sites^19^. The length of the sugar chains influenced the lectin-carbohydrate interactions and the terminal and subterminal sugars affected lectin binding^20^. Several studies showed that the Gal epitope was still expressed in *GGTA1* deficient mice and pigs when stained with anti-Gal alpha(1, 3)Gal mAb or sensitized human serum^9,12,13^, but not IB4 lectin.

To determine whether *iGb3S* deletion affects the immunological properties of *iGb3S* KO mice, antibody titers in *iGb3S* KO mice were analyzed. We did not find any significant differences in total IgG, IgM, and IgA titers in *iGb3S* KO mice when compared with WT mice. Furthermore, RRBC immunization did not induce significant changes in total IgG, IgM, and IgA in *iGb3S* KO mice. However, IgG2b was significantly increased after RRBC immunization in *iGb3S* KO and WT mice, and the increase was greater in *iGb3S* KO mice compared with WT mice. IgG2 is the major IgG form specific for the Gal epitope^21^, and an *in vitro* study showed that the most abundant anti-SIS (a commercial regenerative medical product) antibody subtype that bound to SIS following exposure to human plasma was IgG2^22^. In the present study, higher levels of IgG2 were observed in *iGb3S* KO mice compared with WT mice after RRBC immunization, might suggesting *iGb3S* KO mice are more sensitive to RRBC stimulation compared with WT mice.

Anti-Gal specific antibodies did not be detected in *iGb3S* KO mice (Fig. 3c), even after exogenous immune challenge with RRBC membrane injected intraperitoneally. An explanation might be that because *GGTA1* is the major gene for synthesizing Gal epitope and *iGb3S* gene KO did not affect *GGTA1* mRNA expression (Fig. 3b), therefore *iGb3S* KO mice still express large Gal epitope (only decreased about 5.19% to 21.74% in the main organs), so that the *iGb3S* KO mice could not produce anti-Gal antibodies. These results suggest that the *iGb3S* gene might have a weak effect on the immunological properties of mice. A previous study demonstrated that silencing the porcine *iGb3S* gene did not affect measures of anticipated pig-to-human and pig-to-primate acute rejection, suggesting *iGb3S* is not a contributor to antibody-mediated rejection in pig-to-primate or pig-to-human xenotransplantation^23^. But, they confirmed that *iGb3S* gene silencing significantly changed the renal glycosphingolipid profile. Therefore, whether *iGb3S* deficiency causes other changes to the glycosphingolipid profile and what effects it has on the biological functions of animals requires further investigation.

In conclusion, we generated an *iGb3S* KO mouse model. Our data suggest that the *iGb3S* gene likely contributes to Gal epitope expression, but that *iGb3S* deletion alone did not significantly change immunological properties, implying *iGb3S* deletion alone might have a weak effect on the immunological properties in mice.

Our further study have verified that the Gal antigen expression was decreased by 97.5–99.6% in the main organs of *GGTA1* KO mice (the manuscript is under-reviewing by Journal of Applied Genetics), and *GGTA1* and *iGb3S* double KO in mice generated a completely disappearance in Gal antigen expression (the manuscript is under-drafting). The data from our study suggested that both *GGTA1* and *iGb3S* are the regulators for Gal antigen expression. The detail relationship between *GGTA1* and *iGb3S* for the regulation of Gal antigen expression is investigating in our laboratory.

## Materials and Methods

All animals used in this study were housed and handled in accordance with the guidelines set by the Association for the Assessment and Accreditation of Laboratory Animal Care. The protocol of this study was approved by the National Institutes for Food and Drug Control (NIFDC) Institutional Animal Care and Use Committee. All methods were performed in accordance with the relevant guidelines and regulations of Good Laboratory Practice in NIFDC quality system.

### Animals

C57BL/6 mice (black) and Kunming (KM) mice (white) were purchased from the Institute for Laboratory Animal Resources of NIFDC, and together with *iGb3S* KO mice were maintained in a specific pathogen-free facility at a temperature of 23 ± 1 °C, relative humidity of 30–70%, and a 12-h light/12-h dark cycle.

### Generation of *iGb3S* deficient mice

The *iGb3S* deficient mouse model was generated with support from Beijing Biocytogen (Beijing, China). Briefly, homology regions covering 1.4-kb upstream of exon 1 and 7.5-kb downstream of exon 5 of the *iGb3S* gene were subcloned from a bacterial artificial chromosome (BAC) clone (RP23-241C3; Invitrogen) from the C57BL/6 J mouse genomic RPCI-23 BAC Library.

The targeting vector was constructed by replacing a 1.5-kb fragment encoding the *iGb3S* exon 5 with a loxP-flanked neomycin-resistance gene cassette (neo), and a Diphtheria toxin A (DTA) gene driven by the herpes simplex virus thymidine kinase (TK) promoter inserted into the genomic fragment for negative selection. The two homologous arms, 8.3-kb 5′-arm immediately upstream of the neo cassette and the 9.1-kb 3′-arm immediately downstream of the neo cassette are shown in Fig. 1. The primers used in target vector construction are shown in Table 2.

After linearization, the targeting vector was electroporated into E14 ES cells, and 200 G418-resistant clones were picked, expanded, and characterized by Southern blot analysis using the 5′-external probe and 3′-external probe (data not shown). Of eleven positive ES cell clones, five were microinjected into C57BL/6 blastocysts. Chimeric offspring were backcrossed to C57BL/6 mice and germline transmission was confirmed by PCR of tail genomic DNA (data not shown). Heterozygous F1 progenies were intercrossed to obtain *iGb3S* KO homozygous mice. The mutant mice were kept under specific pathogen-free conditions.

### Southern blot analysis

Genomic DNA isolated from the mutant offspring were digested with *Nde*I (NEB), separated on a 1% agarose gel, and transferred to a positively charged nylon membrane (Hybond N+; Amersham International plc, Little Chalfont, Buckinghamshire, UK). The filter was hybridized with a digoxigenin (DIG) labeled probe. Hybridizing bands corresponding to the *iGb3S* gene were detected using the DIG Luminescent Detection Kit (Roche Applied Science Inc. Indianapolis, IN, USA). For probe labeling, 5′-external and 3′-external DIG-labeled probes were prepared by PCR using Taq DNA polymerase and incorporating DIG-11-dUTP according to the manufacturer’s instructions (Roche Applied Science Inc.). The following primers were used to amplify the 5′ external (479 bp) probe:5′-CATTCTGATACTCTGTCAATCATTTC-3′ (forward primer), and 3′-GGATGGACCACCAGAAACTA-5′ (reverse primer). For the 3′-external (521 bp) probe, the following primers were used: 5′-GTATTACTCGTGAACACTCTTGGC-3′ (forward primer) and 3′-GTAGAGTCAGCGCCTCACAG-5′ (reverse primer).

### RNA isolation and RT-PCR

RNA was extracted from the heart, liver, spleen, lung, and kidney of mice (WT and *iGb3S* KO) using the RNAiso plus Kit (TaKaRa, Tokyo, Japan). Then, 0.4 μg of total RNA was reverse-transcribed in 20 μL total volume using a PrimeScript™ RT reagent Kit with gDNA Eraser (Perfect Real Time, TaKaRa), according to the manufacturer’s instructions. RT-PCR was performed with 2 μL of cDNA. *GAPDH* was used as a housekeeping gene for the normalization of the detected gene. The primers used were as follows: *GAPDH* (182 bp, gene accession number: NC_000072.6): 5′-GTTGTCTCCTGCGACTTCA-3′(forward primer) and 5′-TGGTCCAGGGTTTCTTACTC-3′ (reverse primer); *iGb3S* (177 bp, gene accession number: NM_001009819.2): 5′-CACTTTCGACCCTCATGTAGC-3′ (forward primer) and 5′-GGGCGATCCGTAAACACATAGT-3′ (reverse primer); and *GGTA1* (102 bp, gene accession number: NM_010283.3): 5′-ACCGCCCGGATGTTTTGAC-3′(forward primer) and 5′-TGACGTAAAATATGACCCGATGG-3′ (reverse primer).

### Gal antigen determination

Gal antigen expression in the heart, liver, spleen, lung, and kidney tissues in experimental mice (WT and *iGb3S* KO) was determined using a commercial monoclonal anti-Gal mouse antibody (M86, ALX-801-090-1, α-Gal Epitope, mAb. Enzo Life Science, NY, USA) and a Gal antigen quantitative detection kit (70101, Meitan, Beijing San Yao Science & Technology Development Co., Beijing, China) following the manufacturer’s instructions and previous reports^19,24–27^. Briefly, the solid-phase antigen was coated with 100 μL/well of Galα(1,3)Gal-BSA (Gal-BSA) solution (1–2 μg/mL, NGP0203, Dextra Laboratories, READING, UK) in 96-well plates (Nunc, Rochester, NY, USA), and blocked with 200 μL/well of 1% human serum albumin (A8230, Solarbio, Beijing, China). A calibration curve was produced by using the Gal-BSA/Gal-free matrix (Gal antigen-negative biomaterial reference material provided by the National Institutes for Food and Drug Control, China) as a Gal antigen reference material. Briefly, serial of dilutions of Gal-BSA in 2 mg of Gal-free matrix lysate solution were produced. A Gal antigen-positive biomaterial reference material (provided by National Institutes for Food and Drug Control, China) was used as a positive control to monitor the sensitivity of the test system, and a Gal antigen-negative biomaterial reference material was used as a negative control to monitor the specificity of the test system. All test samples (200 μL/sample) were incubated with the primary monoclonal antibody M86 (200 μL, 20-times dilution according to the kit instructions) for 2 h at 37 °C with gentle shaking and then at 4 °C overnight. Before use, the reaction mixtures were centrifuged at 14,000 × g for 30 min at 4 °C, and 100 μL/well of the supernatant containing residual M86 antibody was loaded onto a Gal-BSA pre-coated 96-well plate, incubated for 1 h with gentle shaking at 37 °C in the dark. After washing, 100 μL of the secondary horseradish peroxidase (HRP)-conjugated antibody (goat anti-mouse IgM-HRP, sc-2064, Santa Cruz, CA, USA) diluted 1:2000 in PBS was loaded and incubated for 30 min. Finally, following washing, 100 μL of HRP color development solution (Trimethylbenzene, TMB, SE1005, Biokorad, Beijing, China) was added to each well for 15–30 min at room temperature in the dark and the reaction was stopped by 50–100 μL of 10% H_2_SO_4_. The absorbance was measured by a microplate reader (Spectramax M5, Molecular Devices, CA, USA) at 450 nm.

Our preliminary experiments confirmed that the mass weight of Galα1-3gal-BSA (Gal-BSA) was approximately equal to the BSA molecule by measuring protein content (data not shown). The relative molecular mass of BSA is 66.33 kD and the number of molecules is 9.08 × 10^18^/g. According to the product information, each Gal-BSA molecule contains about 20 Gal epitopes; therefore the number of Gal epitopes in Gal-BSA is about 1.82 × 10^20^/g. Gal epitopes of test sample were calculated relative to the Gal antigen reference material sample (Gal-BSA) as previously described^27^.

### Immunization treatment

Twenty eight-week-old WT (C57BL/6, body weights are about 26–30 g) and thirty-week-old *iGb3S* KO mice (body weights are about 30–36 g) were immunized with RRBC membrane (1 × 10^8^ RRBC) containing sufficient alpha(1,3)Gal epitopes, by intraperitoneal injection (2 week interval for a total of three times). Littermates without treatment were used as comparisons.

### Determination of immunological factors

The serum of experimental animals was collected and tested for the presence of immunological factors. The total IgG was determined by mouse IgG enzyme-linked immunosorbent assay (ELISA) kit (EMC116, Neobioscience, Beijing, China), and the serum was diluted 1:500,000. Total IgM was determined by mouse IgM ELISA kit (88-50470-22, EBioscience, CA, USA) and the serum was diluted 1:150,000. Total IgA was determined by mouse IgA ELISA kit (88-50450-22, EBioscience) and the serum was diluted 1:10,000. Levels of IgG sub-group, include IgG1, IgG2a, IgG2b, and IgG3 were determined by a seven-immunofactor assay kit (EPX070-20815-901, Beijing, China) and the serum samples were diluted 1:10,000.

Anti-Gal antibody detection was performed by ELISA using Gal-BSA as a solid phase antigen. Briefly, 100 μL/well of Gal-BSA (1.0–2.0 μg/mL in carbonate buffer, pH 9.5) was loaded into a 96-well plate for overnight coating, and then washed with PBS-0.05% Tween-20. It was then blocked with 1% human serum albumin (A8230, Solarbio) for 2 h at 37 °C. Following a wash step, serial dilutions of each serum sample were made in phosphate-buffered saline (PBS)-1% human serum albumin (HSA) and added to the pre-coated plates. The plates were incubated for 2 h at 37 °C and then washed three times with PBS-0.05% Tween-20. Then, 100 μL of goat anti-mouse IgG (1:32,000) (IgG-HRP, sc-2005, Santa Cruz, CA, USA), IgM (1:16,000) (IgM-HRP, sc-2064, Santa Cruz) and IgA (1:1000) (IgA–HRP, sc-3793, Santa Cruz) were added into each well for 1 h at 37 °C. After washing, TMB (SE1005, Biokorad) was added and the colorization was stopped using 10% H_2_SO_4_. The OD was read at 450 nm.

### Statistical analysis

Data are expressed as the mean ± SD. Differences were considered significant when p < 0.05.The significance in different groups was determined by one-way analysis of variance.

## Electronic supplementary material

Supplementary Information

## Data Availability

The all of data are available.
