# Supplementary material for: Gal epitope expression and immunological properties in iGb3S deficient mice
Source: Sci Rep. 2018 Oct 18;8:15433. doi: 10.1038/s41598-018-33032-7 (PMC6194060; doi:10.1038/s41598-018-33032-7)
Supplement: Supplementary file 1 — Supplementary Information [file 41598_2018_33032_MOESM1_ESM.pdf]

Gal epitope expression and immunological properties in *iGb3S* deficient mice

Liming Xu[a,c*], Anliang Shao[a#], Xi Wu[b], Susu Liu[b] , Yan Lu[a,c,d] , Changfa Fan[b §]

[a] Institute of Medical Device Control, National Institutes for Food and Drug Control, 102629, Beijing, China

[b] Institute for Laboratory Animal Resources, National Institutes for Food and Drug Control, 102629, Beijing, China

[c] School of Medical Lab Science and Life Science, Wenzhou Medical University, 325035, Wenzhou, China

[d] Subei People`s Hospital of Jiangsu Province, 225001, Jiangsu, China

Supplementary data file:

1. Homologous recombination at the iGb3S locus in C57BL/6 ES cells was confirmed by Southern blotting and 11 mutant ES cell clones were obtained (Figure S1, S2). The raw data are showed below:

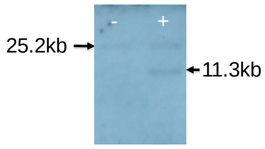

Figure S1. Southern selection by probe1-NdeI. WT: 25.2kb; Mutant: 11.3kb.

As showed in figure S1, the total 12 mutant clones were obtained (plate 1: B1, B11, C7, G11, H6; plate 4: A9; plate 5: C3, C11, D6, E4, G6, G12).

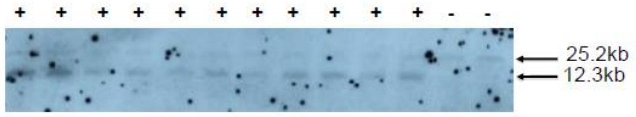

Figure S2. Southern selection by probe2-NdeI. WT: 25.2kb; Mutant: 12.3kb.

As showed in figure S2, among the 12 clones verified by probe 1, total 11 mutant clones (B11, C7, G11, H6, A9, C3, C11, D6, E4, G6, G12) were verified by probe 2.

2. Homozygous iGb3S KO mice were obtained by intercrossing (Figure S3).

The homozygous iGb3S KO mice were obtained by inter-crossing, the photos of Chimeric, Heterozygous and Homozygous iGb3S KO mice are showed below.

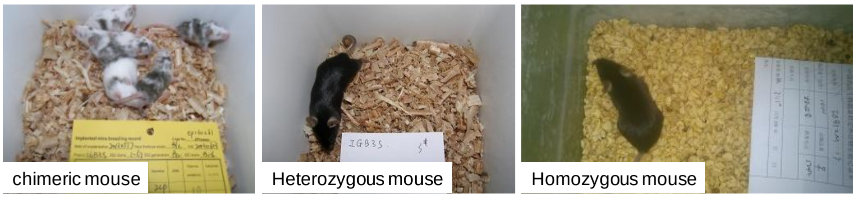

Figure S3. The photos of Chimeric, Heterozygous and Homozygous iGb3S KO mice.

3. Body and organ weights were not significantly different compared with WT littermates (table S1, S2).

There are no significant differences in body weights and some of organ weight in iGb3S KO mice compared to that in WT C57 mice, as showed in table S1 and table S2.

Table S1. The body weight of iGb3S KO mice and WT C57 mice

Old (week)	WT C57 mice body weight (g) (n=6)	iGb3S KO mice body weight (g) (n=6)

14W	27.40 ± 1.67	27.80 ± 1.17
About 18W	29.90 ± 1.50	30.2 ± 1.22

Table S2. The body and organ weight of iGb3S KO mice and WT C57 mice (g)

	Sex		Body weight	Heart	Liver	Spleen	Lung	Kidney	Thymus
WT C57 mice	Female n=6	mean	15.71	0.10	0.79	0.07	0.10	0.20	0.09
		SD	0.64	0.01	0.06	0.01	0.01	0.03	0.02
iGb3S KO mice	Female n=3	mean	15.56	0.10	0.78	0.09	0.11	0.21	0.09
		SD	0.44	0.02	0.13	0.01	0.02	0.03	0.02
	Male n=3	mean	13.95	0.07	0.60	0.06	0.10	0.17	0.07
		SD	0.65	0.01	0.09	0.00	0.01	0.01	0.01

4. Histological examination of main organs did not reveal differences compared with WT littermates (Figure S4)

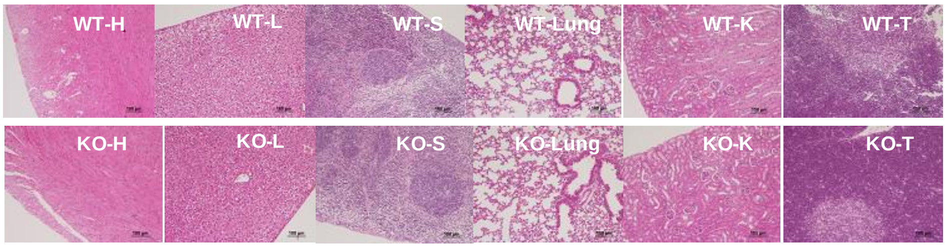

Figure S4. The histological pictures of the main organs in WT C57 mice (WT) and iGb3S KO mice (KO). H: heart; L: liver; S: spleen; K: kidney; T: thymus. (Both WT and KO mice are 4W old, Female)
